# Accidental displacement of a dental implant into the sublingual space: A Case report

**DOI:** 10.4317/jced.52994

**Published:** 2016-10-01

**Authors:** Paolo Cariati, José Fernández-Solís, Ana-Belén Marín-Fernández, Alfredo Valencia-Laseca, Fernando Monsalve-Iglesias

**Affiliations:** 1 Oral and Maxillofacial surgery resident. Hospital Universitario Virgen de las nieves, Granada, Spain; 2 Maxillofacial Surgeon. Hospital Universitario Virgen de las nieves, Granada, Spain

## Abstract

Dental implant surgery is continuously expanding. In fact, every day more and more surgeons are choosing dental implants for allowing great results in the field of oral rehabilitation. However, these procedures are not exempt from complications. This report presents the case of a 66 years old man underwent implant surgery by a specialized dentist. No problems were reported during implant placement. Despite this, three months later, it was displaced into the sublingual space at the time of uncovering. Against this backdrop, the patient was referred to an expert maxillofacial surgeon. Next day, the implant was removed using an intraoral approach to reach the sublingual space. According with our knowledge, there are no cases reported in the literature that describe this complication.

** Key words:**Dental implant, sublingual space, bone atrophy, complications of oral surgery.

## Introduction

The use of dental implant for rehabilitating an edentulous jaw is a common practice (1). The most frequent complications of implant surgery are infection, implant rejection, implant migration and implant rupture (2,3). Interestingly, implant displacement into the maxillary sinus is not an uncommon complication encountered in oral surgery (4,5). Nevertheless, the implant migration into the sublingual space is a complication not reported in the literature. In this line, the displacement of foreign body into this anatomical space might provoke serious complications (6). In fact, it could cause a major infection (7). The aim of the present report is twofold. First, we examine the importance of the proper management of these cases. Second, we describe this rare case with the goal of proposing suitable treatments. Furthermore, we present the radiographic images that were used to locate the implant.

## Case Report

**Figure 1:**
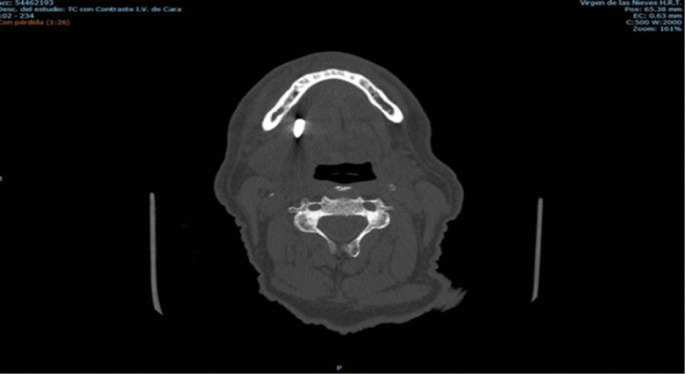
CT images of implant displacement.

**Figure 2:**
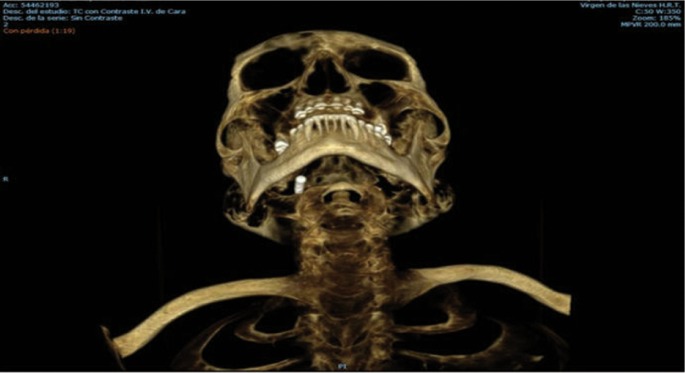
Implant position showed by 3D-reconstruction.

**Figure 3:**
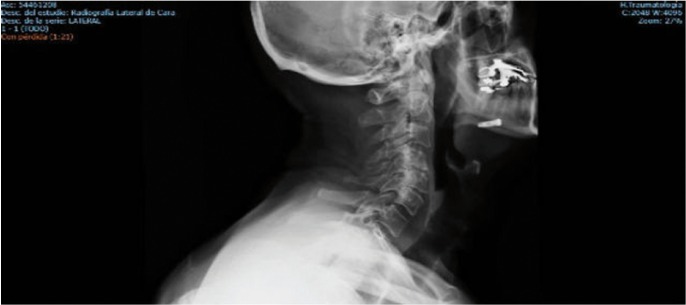
Implant displacement into sublingual space. Side view face X-ray.

We describe the case of a 66-year-old man who underwent implant surgery by a specialized dentist. The aim of surgery was to place a dental implant in the fourth quadrant of the jaw. Is important to note that the patient presented a severe bone atrophy in this sector. No problems were reported during the implant placement. Notwithstanding, few months later, the implant migrated into the sublingual space during the uncovering phase. In light of these facts, the patient was immediately referred to emergency service. Once in the hospital, intravenous antibiotic treatment was administered and a maxillofacial surgeon was contacted. After a careful examinations of the case we decide to remove the implant under general anesthesia and with intraoral approach. The surgery was successful and the patient was discharged from the hospital the day after the procedure, (Figs. 1-3).

## Discussion

The Displacement of a dental implant into the sublingual space is a rare complication of implant surgery. To our knowledge, there no other cases reported in the literature. Regarding the surgical aspects, the present research focused on the importance of a correct management of these cases. Specifically, it is necessary to hospitalize the patient. In fact, is imperative to avoid any manipulation of the sublingual space outside of an operating room. Secondly, the hospitalization is necessary in order to begin an early intravenous antibiotic treatment. Finally, the surgical removal of the implant might be carried out as soon as possible. In fact, the presence of a foreign body into the sublingual space could provoke serious problems. Moreover, we would like to analyze the possible production mechanism of this complication. In fact, the surgical exploration of the patient revealed the presence of a drilling in the inner side of the jaw bone. In view of that, we firmly believe that the implant displacement was caused by the reabsorption of the internal jaw cortical (8). In this sense, we opine that the implant position was initially not adequate to permit the request osseointegration (9). The reason for this is that during the first surgery the implant was severely medially inserted. Consequently, the lack of primary implant stability is the major cause of this complication. Concluding, we would to stress that this report contains two points that are central to us: firstly, numerous variables have to be considered before the beginning of each implant surgery. Specifically, the level of bone atrophy is perhaps the most important parameter. Secondly, we would to stress that the obtaining of great results in advanced dental rehabilitation programs is not a simple task. In this line, we highlight that only expert professionals can effectively perform these procedures.
